# Coronavirus disease 2019 in Saudi Arabia: A nationwide real-world characterization study

**DOI:** 10.1016/j.jsps.2022.02.015

**Published:** 2022-02-25

**Authors:** Khalidah A. Alenzi, Wafi F. Albalawi, Tahani S. Alanazi, Najah S. Alanazi, Deemah S. Alsuhaibani, Nouf Almuwallad, Thamir M. Alshammari

**Affiliations:** a Ministry of Health, Regional Drug Information &Pharmacovigilance Center, Tabuk, Saudi Arabia; b Community Health Sciences, College of Applied Medical Sciences, King Saud University, Riyadh, Saudi Arabia; c Pharm D, University of Tabuk, Tabuk, Saudi Arabia; d Pharm D, Pharmaceutical Care Department, Medical Services for Armed Forces, Ministry of Defense, Riyadh, Saudi Arabia; e King Salman Armed Forces Hospital Northwestern Region: Tabuk, Saudi Arabia; f Saudi Food and Drug Authority, Riyadh, Saudi Arabia; g Medication Safety Research Chair, King Saud University, Riyadh, Saudi Arabia

**Keywords:** Pandemic, COVID-19, Coronavirus, Characteristics, Comorbidities, Diabetes, Hypertension, And intensive care unit

## Abstract

**Background:**

On March 11th, 2020, The World Health Organization (WHO) declared that the COVID-19 is a pandemic due to its worldwide spread. The COVID-19 pandemic has extended its impact to Saudi Arabia. By mid-February 2021, The Kingdom of Saudi Arabia has reported more than 373,000 COVID-19 cases impacting different population categories (i.e., male, female, different age groups, comorbidities status). The objective of this nationwide study was to describe and explore the characteristics of hospitalized patients diagnosed with COVID-19 in Saudi Arabia.

**Methods:**

This study was an observational epidemiological study based on collected clinical data from ten health institutions across all regions in Saudi Arabia. The study was conducted during the period from March 2nd, 2020, to January 31st, 2021. The data were collected included demographics, medical information, medications, and laboratory and diagnostic. More detailed information on usually missing factors such as smoking status, comorbidities, length of hospital stay were also collected. Both descriptive and inferential analyses were conducted using the statistical analysis software “SAS®” version 9.4.

**Results:**

During the study period, 5286 patients were included in this study. Of these, (79.15%) were male. Of all 5286 patients, quite a high number of the studied population 2010 (38.02%) were smokers. The majority of the patients 3436 (65%) were reported to have comorbidities, with hypertension being the most common disease 1725 (32.6%), followed by diabetes 1641(31.04%). A high proportion of the patients, 2220 patients (41.99%), were admitted to the intensive care unit; of these, (33.52%) were on mechanical ventilation. Most patients received anticoagulant prophylaxis medications (n = 4414, 83.5%). All patients were given more than one antibiotic prophylaxis. Overall, the median hospital stay was 5.5 days, and the median length in the intensive care unit was 4.26 days. Around (89.14%) of patients were discharged from the hospital, and (10.8%) died.

**Conclusion:**

In this real-world study utilizing a large sample size, this study provides confirmatory results on the COVID-19 patients characteristics that are similar to other populations. Healthcare professionals need to give COVID-19 patients with specific characteristics including smoking, diabetes mellitus and cardiac disease more care to avoid losing these patients.

## 1 Introduction

On December 31st, 2019, a newly emerging coronavirus was named initially by the World Health Organization (WHO) as (2019-nCoV), and it was considered the cause of the coronavirus disease 2019 (COVID‐19) outbreak (Coronavirus, 2019). Later, because of its similarity with the previous severe acute respiratory syndrome coronavirus (SARS-CoV), it was termed by the International Committee on Taxonomy of Viruses (ICTV) as the severe acute respiratory syndrome coronavirus-2 (SARS-CoV-2) (Coronaviridae Study Group of the International Committee on Taxonomy of, 2020). The outbreaks of several viral diseases have been reported in the last two decades including, the severe acute respiratory syndrome coronavirus (SARS-CoV) in 2002 and the Middle East Respiratory Syndrome Coronavirus (MERS-CoV) in 2012 (Cheng et al., 2007, Zumla et al., 2015). Approximately the symptoms of COVID-19 appear five days after the incubation period and continue for at least 11.5 days (Lauer et al., 2020, Shereen et al., 2020). Moreover, the symptoms vary from gastrointestinal symptoms such as anorexia, diarrhea, and vomiting to neurological manifestations confusion, dizziness, and fever with myalgia (Adhikari et al., 2020). Also, severe complications like Septic shock and multi-organ failure can result from a severe COVID-19 pulmonary infection (Chen et al., 2020c). Early infected cases with COVID-19 had a history of exposure to the Seafood Market in Wuhan, China which indicated animal-to-person transmission. Later, human-to-human transmission via droplets through cough or sneezing was also included (Adhikari et al., 2020).

The Chinese healthcare authorities reported the first known case of Covid 19 in December 2019 (World Health Organization, 2020a, Jun 20). Then, WHO officially announced in March 2020 that Covid 19 is a severe pandemic and global public health emergency because of the widely reported cases worldwide (World Health Organization, 2020b, March 11). The last epidemiological update on Covid-19 has estimated about 3 million reported cases globally and 72,000 new deaths (World Health Organization, 2021, Jun 15) Several Countries have encountered significant challenges on the best management of the Covid 19 outbreak due to the insufficiently published data on disease characteristics, including spreading rate, mode of transmission, mortality rate, etc. Consequently, ineffective management approaches of the Covid 19 pandemic have resulted from the improper understanding of disease characteristics in many healthcare institutions worldwide (Khan et al., 2021).

COVID-19 pandemic adversely affects the economies and millions of people worldwide. Consequently, it caused a significant burden and challenged health care systems in many countries to develop optimal guidelines for surveillance, prevention, and management of the disease. In Saudi Arabia, the first confirmed case was reported on March 2nd, 2020 (Arab News, 2020, March 03). Saudi Arabia has adopted substantial precautionary preventive measures and proactive response to the containment of the COVID-19 pandemic (Alshammari et al., 2020). The marked response in the current COVID-19 pandemic came from the crucial lesson learned from the outbreak of MERS-CoV in 2012 (Obied et al., 2020).

According to literature evaluation, assessment of patients' characteristics has not been evaluated on a large sample size yet. Therefore, this study is important to provide comprehensive data that address the possible variations in patients' characteristics, assess the utilization of the different medications used, optimize treatment based on patient's characteristic, and manage similar pandemic in the future. Therefore, the study aimed to characterize the hospitalized patients with COVID-19 in Saudi Arabia using a large sample size.

## 2 Methods

### 2.1 Study design and data source

The study was a retrospective, observational epidemiological study using collected data for hospitalized patients with COVID-19 from ten institutions in eight different regions (out of 13 regions) in Saudi Arabia.

The study period was between March 2nd, 2020, and ended on January 30th, 2021. Routine care data from included patients were extracted from the patients' medical records, manual records and entered anonymously into a web-based electronic form (Google form). The patient data collected the following variables: patient's demographic (e.g., age, gender, and geographic region), admission date, discharge date, and smoking status). Additionally, medical information (e.g., comorbidities, length of hospital stay, mode of transmission, and hospitalization) was collected. Furthermore, use of supporting care devices (mechanical ventilation, mechanical venous thrombolytic devices), medications including (e.g., antivirals, antimalaria, antifungals, antibiotics, anticoagulants, analgesics, immunoglobulin, Angiotensin Receptor Blockers (ARBs), Angiotensin Converting Enzyme Inhibitors (ACEIs), and other prescribed medications) were also collected. Patients with COVID-19 were defined if they had a confirmed positive polymerase chain reaction (PCR).

We compared patients who died due to COVID-19 during admission and those who were discharged from the hospital (based on the medical record) to assess the predictors for the outcomes.

### 2.2 Statistical analysis

Demographic and clinical characteristics for our study's population were analyzed using chi-square or Fisher's exact tests for categorical data and Mann-Whitney *U* test for continuous data. Utilizing univariable logistic regression analysis, we only included the predictors (variables) that were statically significant associated with the outcome (death) in our final model. Logistic regression was used to compute the odds ratios (ORs) and 95% confidence intervals (CIs) to evaluate the associations between the potential predictors with the outcome.

The data included age (grouped as 18–29, 30–44, 45–64 and 65+ years for descriptive analysis), sex, smoking status. We also considered the following comorbidities: diagnosed hypertension, diabetes, cancer, kidney disease, liver disease, heart disease, epilepsy, autoimmune disease (rheumatoid arthritis/lupus/psoriasis), and chronic respiratory diseases including asthma and COPD. For all analyses, a 2-tailed test with a p-value < 0.05 was considered statistically significant. We calculated variables as percentages from the total population. While we calculated the length of hospital and Intensive Care Unit (ICU) stay among discharged and dead patients by dividing in total discharge and total death. Also, we calculated the ICU length of stay and use of the ventilation and general characteristics of this patient's odds of mechanical ventilation by dividing the number of patients for each period by the total number who used the mechanical ventilation.

The data were managed and analyzed using Statistical Analysis of software (SAS®) (version 9.4, SAS Institute Inc., Cary, NC, USA). We excluded people with missing age, sex outcomes since these are likely to indicate poor data quality.

## 3 Results

In total, 5286 adult patients admitted with COVID-19 were identified. We analyzed the distribution of COVID-19 patients admitted to hospital, discharged, reported dead, and those admitted to ICU across all regions in Saudi Arabia. Eastern region has the highest rate of admitted COVID-19 patients (n = 1147, 21.69%), followed by, Asir region (n = 1043, 19.73%), Makkah region (n = 750, 14.18%) and Riyadh region (n = 679, 12.84%), respectively, Table 1.

**Table 1:** Distribution of COVID-19 Cases across Saudi Arabia.

Region	Total: 5,286	%	Discharge: 4,712	%	Death:574	%	ICU:2,220	%
Eastern region	1147	21.69%	1046	22.19%	101	17.58%	918	41.35%
Asir region	1043	19.73%	957	20.30%	86	15%	664	29.91%
Makkah region	750	14.18%	644	13.67%	106	18.45%	49	2.20%
Riyadh region	679	12.84%	624	13.25%	55	9.58%	242	10.90%
Tabuk region	520	9.83%	488	10.36%	32	5.57%	84	3.78%
Jizan region	432	8.17%	389	8.26%	43	7.49%	90	4.054%
Medina region	367	6.94%	269	5.70%	98	17.1%	76	3.42%
Al-Qassim region	320	6.05%	267	5.67%	53	9.23%	82	3.69%
MISSING	28	0.52%	28	0.59%	0	0%	15	0.67%
TOTAL	5,286	100%	4,712	100%	574	100%	2,220	100%

As for the discharged patients, the highest discharge rate was in the Eastern region (n = 1046, 22.195), followed by Asir region (n = 957, 20.30%) and Makkah region (n = 644, 13.67%), respectively. Out of 574 patients who died, the Makkah region had the highest death rate, (n = 106, 18.45%), followed Eastern region (n = 101, 17.58%), and Madinah region (n = 98, 17.1%), respectively, Table 1

The majority of the studied population was male gender (n = 4,181, 79%), and the mean age was 54. As age groups, the majority of the study population age group was 45–65 (n = 2343, 44%), followed by more than 65 years (n = 1348, 25.5%). Table 2.

**Table 2:** Baseline Characteristics of Patients Hospitalized With COVID-19.

Variables	Relationship with death and discharge				Hospital Stays (N=5286)								Total	
	Discharge (N=4,712)		Death (N=574)		Ward (N=3,066)				ICU (N=2,220)					
	N	%	N	%	Discharge(N=2,848)		Death(N=218)		Discharge(N=1,864)		Death(N=356)		N = 5286	%
					N	%	N		N	%	N	%		
Groups of age (years)														
18 - 29	241	4.56	15	0.28	188	3.56	0	0	53	1.00	15	0.28	256	4.89
30 - 44	1190	22.51	102	1.93	876	16.57	2	0.038	314	5.94	100	1.89	1292	24.44
45 - 64	2091	39.56	252	4.77	1300	24.59	33	0.62	791	14.96	219	4.14	2343	44.32
≥ 65	1146	21.68	202	3.82	599	11.33	14	0.26	547	10.35	188	3.56	1348	25.50
Gender														
Male	3688	69.77	493	9.33	2731	51.66	70	1.32	957	18.10	423	8.00	4181	79.1
Female	1028	19.45	81	1.53	905	17.12	19	0.36	123	2.33	62	1.17	1109	20.98
Smoking	1727	32.67	283	5.35	1717	32.48	38	0.72	10	0.19	245	4.63	2010	38.02
Supporting Therapy														
Mechanical ventilation	1554	29.4	218	4.12	0	0	0	0	1554	29.4	218	4.12	1772	33.52
Pneumatic compression devises	230	4.35	2	0.04	30	0.57	0	0	200	3.78	2	0.04	232	4.39
Comorbidities	2940	55.62	496	9.4	490	9.27	73	1.38	2450	46.35	423	8.00	3436	65
Hypertension	1527	28.89	198	3.74	247	4.67	58	1.1	1280	24.21	140	2.65	1725	32.6
Diabetes mellitus	1388	26.26	253	4.79	401	7.59	2	0.04	987	18.67	251	4.75	1641	31.04
Heart disease	328	6.21	116	2.19	225	4.26	6	0.11	103	1.95	110	2.08	444	8.4
Asthma	233	4.41	22	0.41	171	3.23	0	0	62	1.17	22	0.42	255	4.82
Cancer	33	0.62	46	0.87	6	0.11	1	0.02	27	0.51	45	0.85	79	1.5
Kidney disease	112	2.12	23	0.44	68	1.29	2	0.04	44	0.83	21	0.4	135	2.56
Obese	183	3.46	23	0.44	74	1.4	4	0.08	109	2.06	19	0.36	206	3.89
Anemia	75	1.42	2	0.04	75	1.42	0	0	0	0	2	0.04	77	1.46
Benign prostatic hyperplasia	54	1.02	12	0.23	50	0.95	0	0	4	0.1	12	0.23	66	1.25
Ischemic stroke	52	0.98	30	0.57	23	0.44	1	0.02	29	0.55	29	0.55	82	1.55
Epilepsy	47	0.89	17	0.32	40	0.76	0	0	7	0.13	17	0.32	64	1.21
Autoimmune disease	31	0.59	3	0.06	31	0.59	0	0	0	0	3	0.057	34	0.63
Therapies														
Plasma	207	3.92	16	0.30	22	0.42	1	0.019	185	3.5	15	0.28	223	4.22
Azithromycin	3060	57.89	352	6.66	1786	33.79	120	2.27	1274	24.10	232	4.39	3412	64.55
Linezolid	296	5.6	44	0.83	15	0.28	3	0.057	281	5.32	41	0.78	340	6.43
Ceftriaxone	4438	83.95	471	8.91	1768	33.45	178	3.37	2670	50.51	293	5.54	4909	92.87
Tazocin	610	11.54	95	1.8	200	3.78	32	0.61	410	7.76	63	1.19	705	13.34
Levofloxacin	398	7.53	77	1.46	190	3.59	12	0.23	208	3.93	65	1.23	475	8.99
Vancomycin	647	12.24	74	1.4	120	2.27	22	0.42	527	9.97	52	0.98	721	13.64
Colistin	182	3.44	19	0.36	68	1.28	3	0.06	114	2.16	16	0.30	201	3.80
Meropenem	671	12.69	54	1.02	64	1.21	12	0.23	607	11.48	42	0.79	725	13.7
Dexamethasone	2027	38.35	342	6.47	206	3.89	3	0.06	1821	34.45	339	6.41	2369	44.82
Prednisolone	136	2.57	14	0.26	124	2.34	2	0.038	12	0.23	12	0.23	150	2.84
Hydrocortisone	222	4.2	23	0.44	30	0.57	0	0	192	3.63	23	0.44	245	4.64
Heparin	953	18.03	113	2.14	150	2.84	14	0.26	803	15.19	99	1.87	1066	20.17
LMWH	3048	57.66	359	6.79	1230	23.27	22	0.41	1818	34.39	337	6.38	3407	64.45
Paracetamol	3667	69.37	222	4.2	1900	35.94	4	0.08	1767	33.43	218	4.12	3889	73.57
Favipiravir	1928	36.47	149	2.82	698	13.20	87	1.65	1230	23.27	62	1.17	2077	39.29
Hydroxychloroquine	1316	24.9	92	1.74	1166	22.1	46	0.87	150	2.84	46	0.87	1408	26.64
Interferon-B1	780	14.76	58	1.1	20	0.38	2	0.038	760	14.38	56	1.06	838	15.85
lopinavir \Ritonavir	1411	26.69	96	1.82	376	7.11	33	0.62	1035	19.58	63	1.19	1507	28.51
Tocilizumab	632	11.96	35	0.66	2	0.04	3	0.05	630	11.92	32	0.61	667	12.62
IVIG	67	1.26	8	0.15	5	0.1	0	0	62	1.2	8	0.15	75	1.42
ACE inhibitors	340	6.432	26	0.49	267	5.1	10	0.19	73	1.38	16	0.30	366	6.92
ARBs	191	3.61	13	0.25	123	2.33	2	0.038	68	1.29	11	0.21	204	3.86

Angiotensin Receptor Blockers (ARBs), Angiotensin Converting Enzyme Inhibitors (ACEIs), intravenous immunoglobulin (IVIG), Low-molecular-weight heparins (LMWH)

Four thousand seven hundred and twelve patients (n = 4,712, 89%) were discharged from the hospital, while quite a high number of the patients (n = 574, 10.85%) died in the hospital. A high proportion of the patients, 2220 patients (41.99%), were admitted to the intensive care unit. The median of hospital stays [IQR] was (5.5) days with mean [SD] was 9.18 days, While the median of intensive care stay was (4.26) days with mean [SD] was 7.94 days. High number of patients have comorbidities (n = 3436, 65%), with hypertension being the most common disease (n = 1725, 32.6%), followed by diabetes (n = 1641, 31.04%). and coronary heart diseases (n = 444, 8.4%). On the other hand, only (n = 206, 3.89%) of the patients were obese, Table 2.

The highest mortality rate was (n = 493, 9.33%) among males compared to (n = 81, 1.53%) among females. Furthermore, recovery was higher among the 45–65 age group (n = 2091, 39.6%), followed by the 30–45 age group (n = 1190, 22.51%) from the total population (n = 5286).

Two thousand and ten patients were smokers, which represent (38%) of the studied population, Table 2.A Quite a high number (n = 1772, 33.52%) of patients needed mechanical ventilation. General characteristics of this patient's odds of mechanical ventilation are as follow, Male sex (n = 1312, 74.06%), older age ≥ 45(n = 1428, 80.6%), and the presence of comorbidities (n = 1390, 78.4%) such as diabetes, hypertension, coronary heart diseases, and asthma among the most important of these diseases (46.8%, 44%, 14.6%, 8.5%, respectively). of all patients, (n = 4414, 83.5%) were on anticoagulant prophylaxis medications, including (n = 1066, 20.17 %) on heparin and (n = 3407, 64.45%) on Low-molecular-weight heparin (LMWH) with less than 0.1% on oral anticoagulants and (n = 232, 4.388%) using pneumatic compression devices. Dexamethasone is the most common drug given to patients, followed by favipiravir then hydroxychloroquine (44.82%, 39.29 %, 26.63 respectively). Remdesivir was the lowest prescribed with a percentage not exceeding (n = 2, 0.02%). (n = 2027, 38.4%) of patients treated with dexamethasone and (n = 1928, 36.5%) who took favipiravir were discharged from hospital, as well as (n = 149, 2.8 %) from patients who took favipiravir died. Only (n = 223, 4.22%) took Convalescent plasma therapy with a high discharge rate (n = 207, 3.9%), and (n = 75, 1.42%) took Intravenous Immunoglobulin (IVIG) with a discharge rate (n = 67, 1.2%). (570) patients hospitalized with COVID-19 and who took Angiotensin Converting Enzyme Inhibitors (ACEIs) or Angiotensin Receptor Blockers (ARBs) before hospital admission had a significantly lower risk of death (n = 39, 0.74%).

All patients were given more than one antibiotic prophylaxis, and Ceftriaxone was the most prescribed antibiotic with (n = 5286, 92.87%), followed by Azithromycin with (n = 3412, 64.55%) then Piperacillin/tazobactam, and Meropenem. Moreover, (n = 297, 5.5 %) were given antifungals. Overall, (n = 2232, 42%) of the patients had a duration of stay of seven days, and (n = 1577, 29%) of them had a duration of more than one to two weeks, with a discharge rate of (n = 2033, 38.5%) and (n = 1403, 26.5%), respectively. (n = 1215, 22.9%) of the patients had a duration of stay in ICU less than seven days with a discharge rate of 18.7%. The highest age group admitted to hospital was ≥ 45–<65 years (n = 2343, 44.73%), (n = 935, 17.85%) of these patients had a length of stay ranging from 1 to 7 days. Table 3 and Table 4. While the most prolonged period for most patients in intensive care who used mechanical ventilators was from one to seven days (n = 733, 41.36%), predicted death stood at 12.3% versus 87.69 % discharged from patients who used mechanical ventilation. Table 5.

**Table 3:** Length of hospital and ICU stay among discharged and dead patients:

Variables	Relationship with death and discharge					
Total Hospital stays	Discharge [N = 4712]	%	Death [N = 574]	%	Total [ N = 5286]	
1–7 days	2033	43.15	199	34.67	2232	42.22
8–14 days	1403	29.78	174	30.31	1577	29.83
> 14 days	1279	27.14	201	35.02	1480	27.99
ICU stays						
1–7 days	989	20.99	226	39.37	1215	22.99
8–14 days	431	9.15	78	13.59	509	9.63
> 14 days	444	8.4	52	9.06	553	10.46
	**Hospital stays**	**ICU stays**				
Median [IQR]	5.5	4.26				
Mean [SD]	9.18	7.94				

Intensive Care Units (ICU)

**Table 4:** Length of hospital stay estimates with groups of age.

Groups of age (years)	1–7 days Frequency (Percent) Row Pct Col Pct	>7 - ≤14 days Frequency (Percent) Row Pct Col Pct	> 14 days Frequency (Percent) Row Pct Col Pct	Total Frequency (Percent)
Total Hospital Stay*				
18–29	155 (2.96%) 60.55 7.03	60 (1.15%) 23.44 3.84	41 (0.78%) 16.02 2.79	256 (4.89%)
30–44	579 (11.05%) 44.81 26.25	406 (7.75%) 31.42 26.01	307 (5.86%) 23.76 20.87	1292 (24.67%)
45–64	935 (17.85%) 39.91 42.38	721 (13.76%) 30.77 46.19	687 (13.12%) 29.32 46.70	2343 (44.73%)
≥ 65	537(10.25%) 39.87 24.34	374 (7.14%) 27.77 23.96	436 (8.32%) 32.37 29.64	1347 (25.72%)
ICU Stay**				
18–29	49 (2.18%) 4.08 62.82	12 (0.53%) 2.40 15.38	17 (0.76%) 3.09 21.79	78 (3.51%)
30–44	228 (10.14%) 19.00 54.81	93 (4.14%) 18.64 22.36	95 (4.22%) 17.27 22.84	416 (18.73%)
45–64	528 (23.48%) 44.00 52.28	212 (9.43%) 42.48 20.99	260 (11.4%) 49.09 26.73	1010 (45.49%)
≥ 65	378 (16.56%) 32.92 53.02	182 (8.09%) 36.47 24.43	158 (7.00) 30.55 22.55	716 (33.1%)

* Sample Size = 5286, Frequency Missing = 51

** Sample Size = 2220 Frequency Missing = 32

Intensive Care Units (ICU)

**Table 5:** Length of intensive care unit stay estimates and use of mechanical ventilation.

**ICU stay**	**Mechanical** V**entilations [N = 1772] Frequency** **Percent**		
	**Discharge**	**Death**	**Total**
**1**–**7 days**	635 (35.83%)	98 (5.53%)	733 (41.36%)
**8–14 days**	491 (27.7%)	85 (4.79%)	576 (32.50%)
**> 14 days**	428 (24.15%)	35 (1.97%)	463 (26.12%)
**Total**	1554 (87.69%)	218 (12.3%)	1772 100

Intensive Care Units (ICU)

The logistic regression analyses found that patients with heart diseases (Odds Ratios (OR) = 3.297, 95% confidence intervals (CIs = 1.976–5.501)) hypertension (OR = 1.95, 95% CIs = 1.61–2.35), patients with diabetes (OR = 1.35, 95% CIs = 1.10–1.69), smoker patients (OR = 2.29, 95% CI = 1.86–2.83) COVID-19, patients staying the hospital for more than 14 days (OR = 1.56, 95% CIs = 1.21–2.01), patients staying the ICU for 1–7 days and more than 7 to 14 days (OR = 3.59, 95% CIs = 2.87–4.49) and (OR = 2.38, 95% CIs = 1.73–3.27), respectively, were predictors of mortality in patients with COVID-19. Male gender and old age group were also associated with mortality in patients with COVID-19, however, the association was not statistically significant. Table 6.

**Table 6:** Predictors of mortality among COVID-19 patients.

Variables	OR [CI 95%]
Groups of age (years)	
30–44	1.261[0.742–2.141]
45–64	1.356[0.814–2.259]
≥ 65	1.604[0.952–2.703]
Male	1.116[0.847–1.471]
Smoking	2.293[1.856–2.833]
Mechanical ventilation	0.981[0.808–1.192]
Hypertension	1.947 [1.610–2.354]
Diabetes mellitus	1.358[1.095–1.685]
Heart disease	3.297[1.976–5.501]
Hospital stays	
1–7 days	1.196 [0.945–1.515]
8–14 days	1.189 [0.942–1.5021]
> 14 days	1.563 [1.217–2.006]
ICU stays	
1–7 days	3.590 [2.870–4.490]
8–14 days	2.377 [1.729–3.268]
> 14 days	1.277 [ 0.876–1.863]

Odds ratios (ORs), 95% Confidence Interval (95% CI), Intensive Care Units (ICU)

## 4 Discussion

We have investigated the COVID-19 patients characteristics from a considerably large size data including over five thousand patients from ten institutions in nine different regions (out of 13 regions) in Saudi Arabia. The study period covered the duration between March 2nd, 2020, and January 30th, 2021.

Our study finds the mortality rate of 10.85 % is lowest than globally due to several reasons may be due to increased bed capacity in the Intensive care unit (ICU) by 164%, early outpatient intervention through the creation of “fever clinics” in primary healthcare centers in all regions, and provide protocols for COVID-19 that were continuously updated by the ministry of health (Obied et al., 2020, Ramírez-Soto et al., 2021).

We find the highest number of reported death cases in the Makkah region followed by the eastern region then Madinah region was similar to Alissa et al. study that reported the highest number of death cases in the most populated provinces in Riyadh, Jeddah, Makkah, Madinah, and the Eastern province (Alissa et al., 2021).

Smoking history contributes significantly to increase COVID 19 mortality and morbidity, almost half of the death cases in this study were smokers (49.30%). This result is constant with a previous review that shows a higher morbidity risk among smokers and ex-smokers (22% and 46%) (Alqahtani et al., 2020). Even though the exact mechanism connecting smoking to severe COVID 19 is not well known, smoking might impair immunity or increase the presentation of angiotensin-converting enzyme-2 receptors that are associated with severe acute respiratory syndrome (SARS)-coronavirus (Guo, 2020).

The present study analyzed the characteristics of patients with confirmed COVID-19 infections and found that the mortality rate was (85.88 %) among men. Our study confirms the finding of other studies; a study conducted in Peru showed that men had a higher risk of COVID-19 death than women. High incidence rates, mortality, and fatality increased with age and were higher in men 60 years of age or older (Ramírez-Soto et al., 2021). A study in the U.S. concluded that males had significantly higher death rates from COVID-19 than females; 2% and 1% (p = 0.003) respectively (Gomez et al., 2021). Our finding concordance with other studies showing that the male sex has a higher risk of death in hospitalized adults with COVID-19 (Chen et al., 2020b, Peckham et al., 2020). Alissa et al., study also was found that males accounted for 74% of total deaths, and the highest percentage of deaths per age group was between the age of 60 and 69 years (Alissa et al., 2021). Male patients were more vulnerable to severe COVID-19 (Hao et al., 2020, Jin et al., 2020, Richardson et al., 2020); this observation might be attributed to the high presence of many X chromosome genes responsible for solid immunity; the example was defense (Bienvenu et al., 2020). In contrast, a study conducted in India showed that the COVID-19 case fatality rate was higher in women than men; 3.3% and 2.9%, respectively (Joe et al., 2020).

We found that most of the participants were in the age group 45–65, followed by the age group of 65 + years, and the lowest age group admitted was 18 to 30 years. These results are consistent with a study found that the younger population are the least affected group (Burn et al., 2020). A study conducted in Iran showed that the highest incidence of the disease was within the age group of 50–59 years, while the highest mortality rate was within the age range of 70–79 years, among which about 30% confirmed cases died; however, the highest recovery rate was in the 30–39 years age group (65.2%) (Kalantari et al., 2020). A *meta*-analysis includes 59 studies, and more than 36,000 patients found that patients aged 70 years and older have a higher infection risk, a higher risk for severe COVID-19 disease, a higher need for intensive care, and a higher risk of death once infected compared with patients younger than 70 years (Pijls et al., 2021).

This study found that the hospital length of stay was 5.5 days and 4.26 days in ICU. The length of study is one of the essential factors in determining characteristics for patients with COVID-19. Many COVID-19 studies have included length of stay to understand the likelihood and effects of different factors on recovery rate. For example, A systematic review and data synthesis including 52 studies showed that the estimated hospital length of stay for COVID-19 patients varied from less than a week (∼5 days) to nearly two months (Rees et al., 2020). According to the cross-sectional study conducted in a tertiary hospital with comorbidities such as Congestive Heart Failure (CHF), Chronic Obstructive Pulmonary Disease (COPD), Cerebrovascular accident, and End-Stage Renal Disease (ESRD), the length of stay increased in patients with available data from 706 patient (Alwafi et al., 2021). However, we found diabetes and hypertension were the most frequently reported risk factors among dead patients, similar to results of studies that have found that most risk factors for COVID-19 patient deaths were diabetic and hypertensive patients (Gold et al., 2020, Tian et al., 2020). Alissa et al.'s 2021 study found the most common risk factors for death were diabetes (60%), hypertension (50%), chronic renal impairment, and obesity (10%), and our results were similar to it. (Alissa et al., 2021)

In a study conducted in the US with analyzed data from 2491 adults hospitalized with laboratory-confirmed COVID-19, they found a strong direct proportion between age increasing and ICU admission rate; therefore, they consider that a risk factor for ICU admission. In this study, we found that the admission rate to the ICU increased in older age groups more than 45 years compared to those younger (18–45) (Kim et al., 2021).

There is a wide variation in the need for invasive mechanical ventilation in ICU patients among different studies. Moreover, it is associated with a high mortality rate ranging from 16% to 78% (Grasselli et al., 2020, Wang et al., 2020). We find a higher requirement for assisted ventilation observed among male and elderly patients. Furthermore, diabetes and hypertension were the most likely risk factors that led to mechanical ventilation. This wide variation in ICU mortality could be explained by different patient factors, ICU beds capacity, adequate staffing, different organizations, and treatment guidelines. Older age, male sex, history of hypertension, diabetes, chronic obstructive pulmonary disease, and hypercholesterolemia were risk factors with decreased survival in ICU patients (Richardson et al., 2020, Gold et al., 2020).

Approximately 10%–25% of hospitalized patients may develop coagulation dysfunction (Yang et al., 2020). Therefore, Thromboembolic prophylaxis for all hospitalized patients with COVID-19 with LMWH is recommended (Grasselli et al., 2020, Wiersinga et al., 2020). In addition, the American Society of Hematology (ASH) recommends the administration of prophylactic-intensity anticoagulation for COVID-19 patients with respiratory or cardiovascular failure requiring advanced clinical support and for patients who require hospital admission without advanced clinical support (Cuker et al., 2021).

Its predictable effect and convenient use may explain the increased use of LMWH compared to unfractionated heparin (UFH). Also, concerning its route of administration, it is more convenient for bedridden patients with severe symptoms than oral anticoagulants (Levi et al., 2020). In our study, the use of dexamethasone associated with a higher rate of recovered (38.34%), similar to studies conducted studies on COVID-19 patients with moderate to severe Acute Respiratory Distress Syndrome (ARDS), founds that dexamethasone was associated with a significant increase in ventilator-free days, decrease in Sequential Organ Failure Assessment (SOFA), secondary infections, and serious adverse events (Sterne et al., 2020, Tomazini et al., 2020). The beneficial effect of corticosteroids on severe COVID-19 may be attributed to their anti-inflammatory effects on cytokine storms developed in those patients (Rhen & Cidlowski, 2005). One study found that favipiravir was associated with significant improvement in disease progression and viral clearance compared with Lopinavir/Ritonavir (Cai et al., 2020). Moreover, it is associated with a shorter period of fever reduction and cough relief (Chen et al., 2020a).

In this study, few patients used IVIG and were mostly discharged. Thus, the use of IVIG for COVID-19 patients is conflicting. IVIG was partially associated with lower mortality and more extended hospital stay in critically ill patients (Xiang et al., 2021). Although moreover, a Higher dosage of IVIG was a significant factor that led to positive clinical outcomes in COVID-19 cases (Cao et al., 2020), the time of administration was a substantial factor as well, early administration of IVIG in the first 48 h is linked to much lower mortality rate, shorter duration of ICU and reduced use of mechanical ventilation (Xie et al., 2020). However, one *meta*-analysis showed contrary results representing a nonsignificant association of IVIG with lower mortality (Pei et al., 2020). These reported differences in IVIG efficiency may be attributed to the dosage used, administration time, and patients' severity. Also, the plasma therapy shows significant antiviral activity in previous investigations (Li et al., 2020, Ferrario et al., 2005, Li et al., 2020, Sommerstein et al., 2020).

We reported that the third generation cephalosporins (ceftriaxone) was the most antibiotics consuming in COVID-19 patients, followed by azithromycin. Despite the studies that reported a low rate of bacterial/fungal coinfection in patients presenting with COVID-19; however, the consumption of broad-spectrum antibiotics was widely reported, especially in ICU patients (Rawson et al., 2020). A recent rapid review and *meta*-analysis including 154 studies with available data from (30,623) patients out of a total of (35,263) patients showed that the prevalence of antibiotics prescribing was (74.6%). At the same time, estimated bacterial coinfection was (8.6%) from (31) studies (Langford et al., 2021).

In addition, a previous narrative review study found that the Azithromycin is the most frequently used antibiotic (Yacouba et al., 2021), while in another previous study, the 3rd generation Cephalosporins was the most consumed antibiotics (56.7%), followed by Macrolides (22.3%); either in ICUs or other hospital's wards (Mah-E-Muneer et al., 2021).

This study finds the use of ACE or ARB not associated with increased risk of COVID-19 hospitalization or subsequent complications or death, similar to the finding of multinational open science cohort study was conducted in the US and Spain; and this study included more than one million patients (Morales et al., 2020).

## 5 Strengths and limitations

To the best of our knowledge, this is the first epidemiological and clinical characteristics analysis study at the national level representing eight regions in Saudi Arabia with 5286 patients. Furthermore, it provides information on both diseases, hospital stays and therapeutic utilization in patients with COVID-19. However, the study is limited by that; Some patients' profiles are not completed that lead to exclude these patients which might affect our results. Also, there is no information on the exact cause of death.

## 6 Conclusions

This largest real-world study investigated COVID-19 patient's characteristics in Saudi Arabia. It provided insights on different important patient's information including demographic, medical and medications information. Health institutions might benefit from the study results and keep more attention and consideration for smoker patients, patients with heart diseases, patients with diabetes mellitus and patients with hypertension since these patients have the highest risk to die if they have COVID-19 or to follow up with these patients to monitor COVID-19 complications and try to mitigate them

## Declaration of Competing Interest

The authors declare that they have no known competing financial interests or personal relationships that could have appeared to influence the work reported in this paper.
